# A Novel Subpopulation of Prepositus Hypoglossi Nucleus Neurons Projecting to the Cerebellar Anterior Vermis and Hemisphere in Rats

**DOI:** 10.1523/ENEURO.0130-25.2025

**Published:** 2025-07-08

**Authors:** Taketoshi Sugimura, Kazuya Masuda, Yasuhiko Saito

**Affiliations:** Department of Neurophysiology, Nara Medical University, Kashihara 634-8521, Japan

## Abstract

The prepositus hypoglossi nucleus (PHN), involved in horizontal gaze control, contributes to this function via cooperation with the vestibulocerebellum (VC). Furthermore, some PHN neurons have been observed to project to cerebellar regions outside the VC. We previously reported a neuronal population in the ventral caudal PHN that projects to lobules III–V of the anterior vermis or to the cerebellar hemispheric crus. Because the properties of these neurons have not been clarified, this study aimed to determine their localization, projections, and electrophysiological and morphological characteristics in male rats. Tracing experiments revealed that these neurons were clustered within the ventral caudal PHN, approximately between the bregma −12.72 and −12.00 mm, and did not project to the uvula/nodulus (UN), which is part of the VC. Whole-cell recordings and morphological experiments revealed that these PHN neurons exhibited high input capacitance, low input resistance, low-frequency firing, prominent voltage sag, and a multipolar shape. These results indicate that a cluster of neurons in the ventral caudal PHN projecting to lobules III–V of the anterior vermis and hemispheric crus share distinct electrophysiological and morphological properties. Furthermore, these PHN neurons are likely to constitute a distinct subpopulation from PHN neurons projecting to the VC in terms of their projection targets. While previous research has focused on PHN projections to the VC and their role in ocular motor control, this study suggests that this subpopulation may be involved in other motor functions, as the cerebellar anterior vermis and hemisphere are known to contribute to broader motor control.

## Significance Statement

This study examined a subpopulation of neurons in the ventral caudal prepositus hypoglossi nucleus (PHN) that project to the cerebellar anterior vermis and hemisphere, with the properties of these neurons having not been previously characterized. We demonstrated that these neurons exhibit distinct localization, electrophysiological, and morphological properties, thus suggesting that they are different from previously characterized PHN neurons. Our findings suggest that this PHN subpopulation (via its interactions with the cerebellar vermis and hemispheres) may contribute to broader motor control functions beyond oculomotor control and highlight the functional heterogeneity within the PHN.

## Introduction

The prepositus hypoglossi nucleus (PHN) is a brainstem region involved in the control of horizontal gaze (Robinson, 1975, 1989; Fukushima et al., 1992; Fukushima and Kaneko, 1995; Moschovakis, 1997; McCrea and Horn, 2006). PHN neurons encode eye velocity and eye position signals (Delgado-García et al., 1989; Escudero et al., 1992), and those neurons projecting to the abducens nucleus have been reported to primarily convey eye position signals (Escudero et al., 1992). Neural connections between the PHN and the vestibulocerebellum (VC), including the flocculus and the uvula and nodulus (UN), have been shown across experimental species (Blanks et al., 1983; Sato et al., 1983; Langer et al., 1985; Blanks, 1990; Matsuno et al., 2016; Sugimura and Saito, 2021; Blot et al., 2023; Sugimura et al., 2024). The PHN and VC collaborate to control horizontal gaze through a neural network and together form an oculomotor neural integrator that converts transient burst signals proportional to eye or head velocity into sustained signals proportional to eye position (Robinson, 1974; Zee et al., 1981; Godaux and Vanderkelen, 1984; Kheradmand and Zee, 2011; Leigh and Zee, 2015; Beh et al., 2017; Shadmehr, 2017; Shemesh and Zee, 2019).

Neurons in the ventral caudal PHN have been reported to project to the flocculus and nodulus (Brodal and Brodal, 1983; McCrea and Baker, 1985a; Røste, 1989; McCrea and Horn, 2006). However, in our previous study, Sugimura and Saito (2021) reported that when a tracer was injected into distinct cerebellar areas from the VC, namely, lobules III–V in the anterior vermis or the paramedian lobule and crus II, retrogradely labeled neurons with large cell bodies that formed a cluster were observed in the ventral caudal PHN, although some retrogradely labeled neurons were also observed in the rostral and intermediate parts of the PHN. Furthermore, a study using choline acetyltransferase (ChAT)-tdTomato transgenic rats revealed that this population of neurons was not positive for ChAT, indicating that these neurons are glutamatergic. These results suggest that the neuronal population that is located in the ventral region of the caudal PHN and projects to the cerebellar anterior vermis or hemisphere forms distinct prepositocellebellar neurons from the population that projects to the VC.

Although we previously reported the discovery of the population of ventral caudal PHN neurons (Sugimura and Saito, 2021), the precise localization of the population and the physiological, anatomical, and morphological characteristics of the neurons have not been investigated. To determine whether this population forms a prepositocellebellar pathway distinct from the PHN→VC pathway, we systematically investigated the localization of ventral caudal PHN neurons that project to lobules III–V in the anterior vermis (preposito-lobule III–V neurons) or to crus I and II in the cerebellar hemisphere (preposito-crus I and II neurons) and determined whether these neurons also project to the UN, which is part of the VC. PHN neurons are known to be electrophysiologically and morphologically heterogeneous (McCrea and Baker, 1985b; Idoux et al., 2006; McCrea and Horn, 2006; Shino et al., 2008; Kolkman et al., 2011; Zhang et al., 2014; Gaál et al., 2015; Saito et al., 2015; Saito and Yanagawa, 2017). Rat PHN neurons exhibit different firing patterns in response to depolarizing current pulses (Shino et al., 2008, 2011; Saito et al., 2012, 2015, 2017; Saito and Yanagawa, 2013, 2017; Zhang et al., 2014). The firing patterns can be classified into six patterns, including a low firing rate (LFR) pattern that is characterized by a low number of spikes during a depolarizing current injection (despite sufficient membrane depolarization). This classification is useful because these firing patterns reflect unique intrinsic electrophysiological properties and are associated with unique input–output relationships (Shino et al., 2008; Zhang et al., 2014; Saito et al., 2015; Saito and Yanagawa, 2017). Therefore, to examine whether the preposito-lobule III–V and preposito-crus I and II neurons in the ventral caudal PHN show heterogeneous characteristics, we investigated their electrophysiological and morphological properties using whole-cell patch–clamp recordings.

## Materials and Methods

All experimental procedures were approved by the Animal Care Committee of Nara Medical University. The experiments were carried out in accordance with the guidelines outlined by the US National Institutes of Health regarding the care and use of animals for experimental research (ARRIVE guidelines 2.0; Percie du Sert et al., 2020). Eighteen male Long–Evans rats and four male ChAT-tdTomato transgenic rats were used in this study.

### Tracer injection

The tracer injection methods used were similar to those previously described (Sugimura and Saito, 2021; Sugimura et al., 2024). Tracer injections were performed in rats aged 7–8 weeks for histological experiments and rats aged 15 d for electrophysiological experiments. Dextran-conjugated Alexa Fluor 488, Alexa Fluor 594, or Cascade Blue (5% in Tris-buffered saline, Invitrogen/Thermo Fisher Scientific) was injected into the UN, lobules III–V in the anterior vermis, or crus I and II in the cerebellar hemisphere. The tracers were injected unilaterally into crus I and II and into the midline regions of the UN and lobules III–V in the vermis. Under inhalation anesthesia with 2–3% isoflurane, the rats were placed in a stereotaxic apparatus. Using a dental drill, a small hole was drilled in the cranium over the midline region of the posterior vermis, the midline region of the anterior vermis, or the posterior hemisphere for tracer injection into the UN, lobules III–V, or crus I and II, respectively. A glass micropipette with a tip diameter of 40–60 μm was connected to a Hamilton syringe with polyethylene tubing and filled with oil. The tracer was drawn into the tip of the micropipette. For the injections into the three regions, the tip was positioned on the midline, 4.8 mm posterior to the lambda, at a depth of 4.8 mm for the UN; on the midline, 2.3 mm posterior to the lambda, at a depth of 2.0 mm for lobules III–V in the vermis; and 4.5 mm lateral to the midline, 5.0 mm posterior to the lambda, at a depth of 1.2 mm for crus I and II. Tracer injection was performed by applying pressure for >5 min per site. After a total of 0.6–0.8 μl of tracer was injected per rat, the pipette was held in position for 5 min before withdrawal. Following incision suturing, an antibiotic (gentamicin ointment; MSD K.K.) was applied to the wound, and a nonsteroidal anti-inflammatory drug (flunixin meglumine, 1.5 mg/kg; Sigma-Aldrich) was applied subcutaneously.

### Histological procedures and observation

**Figure 1.**
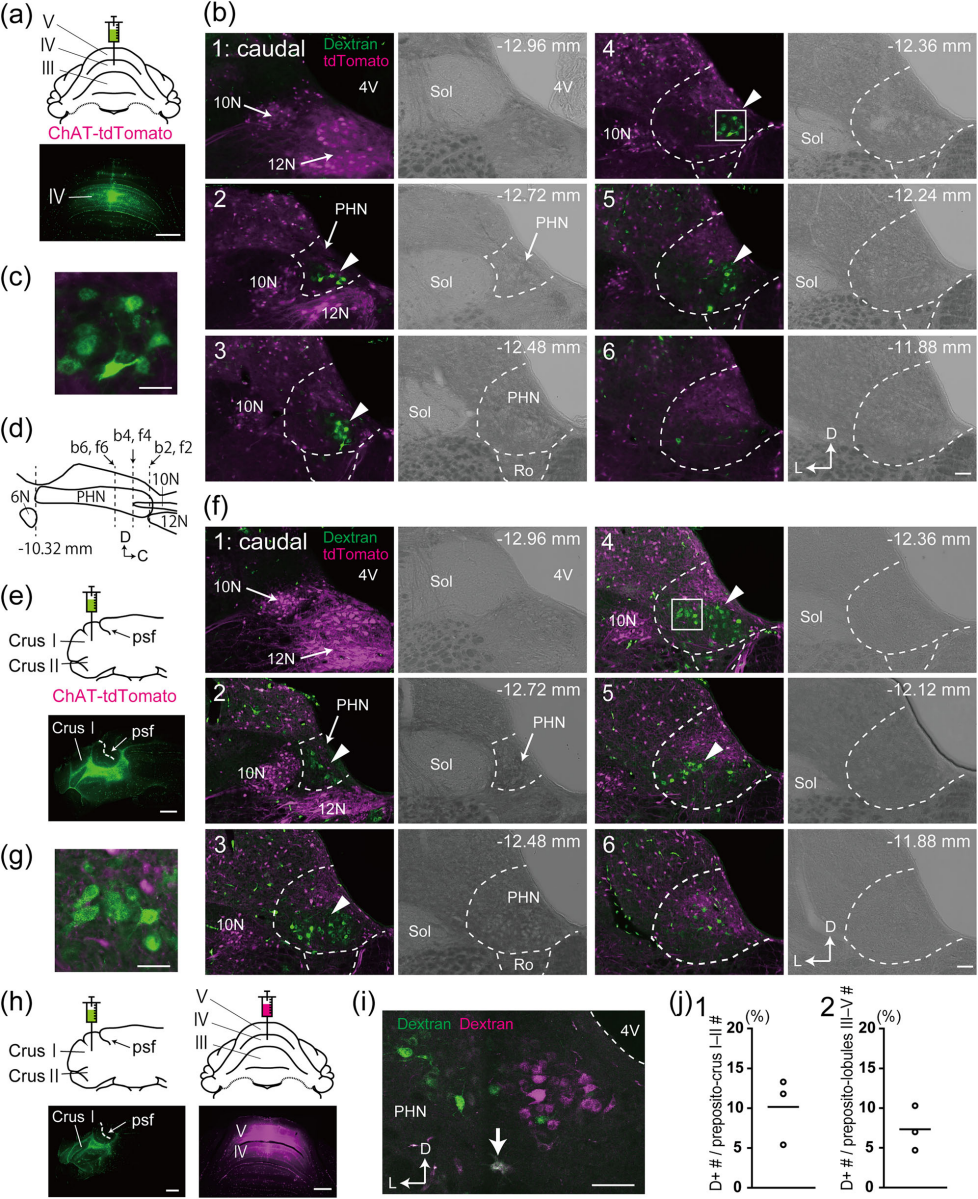
PHN neurons retrogradely labeled via tracer injection into lobules III–V of the vermis and crus I and II of the cerebellar hemisphere. ***a***,***e***, Schematic drawings showing the injection of dextran-conjugated Alexa Fluor 488 into lobules III–V of the vermis (***a***) and crus I and II of the cerebellar hemisphere (***e***) in ChAT-tdTomato rats, along with fluorescence photomicrographs of Alexa Fluor 488 at the injection sites. ***b***,***f***, Fluorescence photomicrographs and bright-field images of frontal sections containing the PHN. The dashed line approximately indicates the boundary of the PHN and the nucleus of Roller. The distance from the bregma is indicated. ***c***,***g***, Enlarged images of the box in ***b*** and ***f***. ***d***, Schematic drawing of a sagittal section through the PHN, with dotted lines indicating the planes of frontal sections shown in ***b*** and ***f***. ***h***, Schematic drawings showing the injection of dextran-conjugated Alexa Fluor 488 into crus I and II and dextran-conjugated Alexa Fluor 594 into lobules III–V of the vermis in wild-type rats, along with fluorescence photomicrographs of the corresponding injection sites. ***i***, A confocal image of a frontal section containing the PHN. The arrow indicates a double-labeled neuron. ***j***, The percentage of double-labeled (D+) neurons among preposito-crus I and II and preposito-lobule III–V neurons in the ventral caudal PHN. Scale bars, (***a***, ***e***, ***h***) 1 mm; (***b***, ***f***, ***i***) 100 μm; (***c***, ***g***) 50 μm. 4 V, fourth ventricle; 6N, abducens nucleus; 10N, dorsal motor nucleus of the vagus; 12N, hypoglossal nucleus; PSF, posterior superior fissure; Ro, nucleus of Roller; Sol, nucleus of the solitary tract. Each Roman numeral indicates the number of the cerebellar lobule. The PSF is shown as an anatomical landmark in photomicrographs to indicate that the injection site was within crus I and II. In photos ***e*** and ***h***, the PSF is indicated by dashed lines.

**Figure 2.**
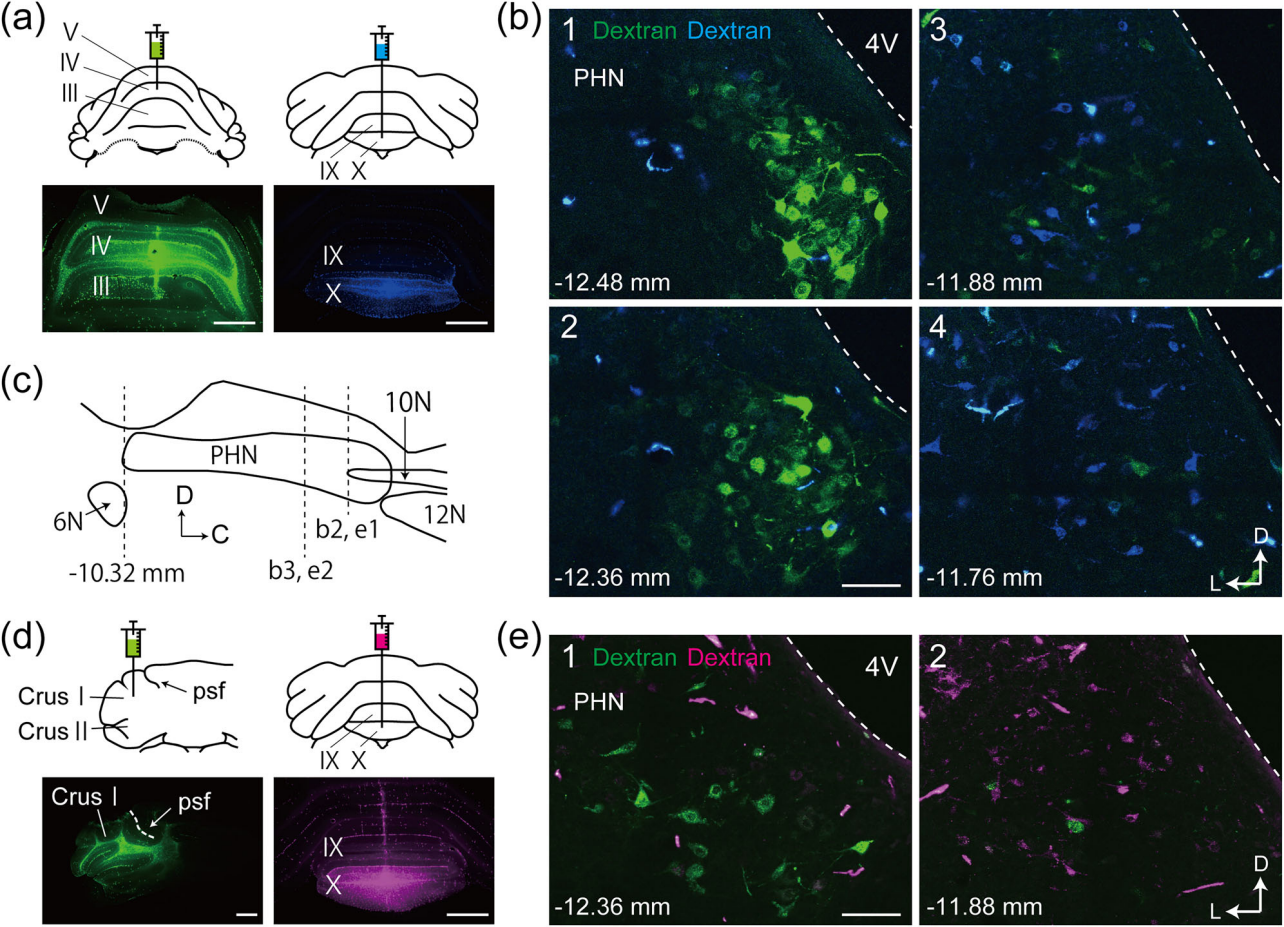
Preposito-lobule III–V and preposito-crus I and II neurons in the ventral caudal PHN did not project to the UN. ***a***, Schematic drawings showing the injection of dextran-conjugated Alexa Fluor 488 into lobules III–V of the anterior vermis and dextran-conjugated Cascade Blue into the UN in a wild-type rat, along with fluorescence photomicrographs of the injection sites. ***b***, Confocal images of frontal sections containing the PHN, with the distance from the bregma indicated. ***c***, Schematic drawing of a sagittal section through the PHN, with dotted lines indicating the planes of frontal sections shown in ***b*** and ***e***. ***d***, Schematic drawings showing the injection of dextran-conjugated Alexa Fluor 488 into crus I and II and dextran-conjugated Alexa Fluor 594 into the UN in a wild-type rat, along with fluorescence photomicrographs of the injection sites. The PSF is shown as an anatomical landmark in the photomicrograph to indicate that the injection site was within crus I and II. ***e***, Confocal images of frontal sections containing the PHN, with the distance from the bregma indicated. Scale bars, (***a***, ***d***) 1 mm; (***b***, ***e***) 100 μm. 4 V, fourth ventricle; 6N, abducens nucleus; 10N, dorsal motor nucleus of the vagus; 12N, hypoglossal nucleus; PSF, posterior superior fissure. Each Roman numeral indicates a cerebellar lobule.

Three to five days after tracer injection, the rats were anesthetized via isoflurane inhalation followed by intraperitoneal injection of a mixture of medetomidine (0.15 mg/kg), midazolam (2.0 mg/kg), and butorphanol (2.5 mg/kg). The rats were then transcardially perfused with 0.01 M phosphate-buffered saline (PBS), pH 7.4, followed by 4% paraformaldehyde in 0.1 M phosphate buffer (PB). The brain was removed and postfixed in the same fixative for 1 d. The injection sites and the brain areas selected for observation were dissected and cut frontally into 60 μm sections via a microslicer (Dosaka EM). The sections were mounted on MAS-coated slides (Matsunami Glass) with antifade medium [Fluoromount (DBS, K024)]. Images of the brain sections were captured under a laser-scanning confocal microscope (C2+, Nikon) or a fluorescence microscope (BZ-X710, Keyence). The images were then processed and analyzed via Fiji (Schindelin et al., 2012). For the tracer experiments for the PHN, every other 60 µm coronal section of the caudal PHN was examined. All coordinates were determined on the basis of the rat brain atlas (Paxinos and Watson, 2018). To precisely localize PHN neurons, we used ChAT-tdTomato transgenic rats, in which cholinergic neurons express the fluorescent protein tdTomato (Zhang et al., 2014). In these rats, the dorsal motor nucleus of the vagus nerve and the hypoglossal nucleus are clearly labeled with tdTomato fluorescence, serving as landmarks for identifying the caudal PHN region. Cell counting was performed using Fiji by applying thresholding to convert images to binary images (threshold set to 65 for an 8 bit image), followed by the “Analyze Particles” function. Retrogradely labeled neurons following tracer injection were automatically counted. To ensure accuracy, particles smaller than 100 µm^2^ were automatically excluded from the analysis, and non-neuron–shaped particles were manually removed. PHN neurons that were double-labeled with tracers were examined within the range of the approximately bregma −12.72 to −12.00 mm using three tissue sections at 180 μm intervals (Figs. 1, 2).

### Slice preparation and whole-cell recording

The slice preparation and whole-cell patch–clamp recording procedures were similar to those previously described (Saito et al., 2015; Saito and Yanagawa, 2017). Briefly, under deep isoflurane anesthesia, a rat (aged 18–21 d) was decapitated, and its brain was quickly removed. Frontal brain slices (250 μm thick) that included the caudal PHN were cut using a microslicer (Pro 7, Dosaka EM) in ice-cold modified extracellular sucrose solution. The slices were recovered in an interface-type chamber perfused with an extracellular solution containing (in mM) 125 NaCl, 2.5 KCl, 2 CaCl_2_, 1 MgCl_2_, 1.25 NaH_2_PO_4_, 26 NaHCO_3_, and 25 glucose and aerated with 95% O_2_ and 5% CO_2_, pH 7.4, at 33°C for 1 h; thereafter, the slices were incubated in oxygenated extracellular solution at room temperature. For recordings, each slice in a submerged recording chamber was continuously perfused with extracellular solution at 3 ml/min. The bath temperature was maintained at 30–32°C using an in-line heater (SH-27A, Warner Instruments). Whole-cell current–clamp and voltage–clamp recordings were performed using fluorescence and infrared differential interference contrast optics with a 40× 0.8 NA water-immersion lens. A MultiClamp 700B amplifier (Molecular Devices) was used, and data were acquired using a pClamp10 system (Molecular Devices). All recordings were obtained from neurons in the ventral caudal PHN. Patch pipettes were filled with an internal solution containing (in mM) 120 K-methylsulfate, 10 KCl, 0.2 EGTA, 2 MgATP, 0.3 NaGTP, 10 HEPES, 10 Na_2_-phosphocreatine, and 0.1 spermine, the pH of which was adjusted to 7.3 with KOH. The osmolarity of the internal solution was 280–290 mosmol/L, and the resistance of the patch electrodes was 3–7 MΩ in the bath solution. The voltage and current signals were subjected to low-pass filtering at 3 kHz and digitized at 10 kHz. The value of the liquid junction potential (−5 mV) was corrected. Upon rupture of the cell membrane, the resting membrane potential was recorded immediately. Neurons with a resting membrane potential below −50 mV and action potential peaks above 0 mV were selected for further analysis. The input capacitance was determined on the basis of the current induced by a 5 mV voltage step from a holding potential of −70 mV using the Membrane Test protocol in pClamp. During current-clamp recordings, the membrane potentials of neurons were maintained between −85 and −75 mV by continuous constant current injection, and depolarizing and hyperpolarizing current pulses (400 ms or 1 s in duration) were subsequently applied. For the analysis of the action potential profiles, including the afterhyperpolarization (AHP) profiles, the depolarizing current pulses were adjusted to induce one action potential in 1 s.

### Post hoc morphological analysis

For morphological analysis, biocytin (0.2–0.3%) was added to the electrode solution to visualize the recorded cells. After whole-cell recording, the slices were fixed with 4% paraformaldehyde in 0.1 M PB, pH 7.4, overnight. Slices without resection were incubated in streptavidin conjugated to Alexa Fluor 568 (1:1,000; S11226, RRID: AB_2315774, Life Technologies) in 25 mM PBS containing 0.1% Triton X-100 overnight at room temperature. The PHN neurons, visualized by streptavidin staining, were imaged via a confocal microscope (20× objective) with a *Z*-axis slice interval of 2.5 µm. Images were acquired from the surface of the slice to a depth where the fibers were no longer visible and were then processed into a *z*-stack. The soma area of biocytin-filled neurons was measured using Fiji by manually tracing the soma boundary with the polygon selection tool and calculating the enclosed area.

### Data analysis

Off-line analysis was performed using the AxoGraph X software (RRID: SCR_014284). The input resistance was estimated on the basis of the voltage response induced by a hyperpolarizing current pulse of −50 pA. The height of the action potential was defined as the difference between the peak of the action potential and the action potential threshold, which was the membrane potential at which the derivative of the voltage trace reached 10 V/s. The half-width of the action potential was defined as the spike width of the half-amplitude from the threshold. The amplitude of the AHP was estimated as the difference between the most negative point of the AHP and the action potential threshold. The sag ratio was calculated on the basis of the voltage response to −300 pA current pulses, expressed as the ratio of B to A (Fig. 5*b*). All values are presented as the means ± SD, with the error bars in the figures representing the SD. In histological experiments using tracers, *n* refers to the number of rats used for the analysis, whereas in electrophysiological and morphological experiments, *n* refers to the number of neurons analyzed. The analyses were performed using GraphPad Prism 8 (GraphPad Software).

## Results

### Clustered ventral caudal PHN neurons projecting to lobules III–V of the vermis and crus I and II of the cerebellar hemisphere

To precisely identify and localize the ventral caudal PHN neurons projecting to the anterior vermis or cerebellar hemisphere, we injected dextran-conjugated Alexa Fluor 488 into lobules III–V of the anterior vermis (*n* = 2; Fig. 1*a*) and into crus I and II of the cerebellar hemisphere (*n* = 2; Fig. 1*e*) in ChAT-tdTomato transgenic rats. The most caudal part of the PHN is wedged between the hypoglossal nucleus (12N) and the dorsal motor nucleus of the vagus nerve (10N) (McCrea and Horn, 2006), both of which were labeled with tdTomato fluorescence (Fig. 1*b*2*,d,f*2), and these two cranial nerve nuclei served as primary anatomical landmarks for identifying neuronal populations within the caudal PHN. In both cases of tracer injection, many retrogradely labeled neurons with large cell bodies were observed as clusters in the ventral caudal PHN (Fig. 1*b*,*f*, arrowheads). These neurons (arrowheads) were located in the ventral caudal PHN and did not correspond with the Roller nucleus, which includes large cells (McCrea and Baker, 1985b; Røste, 1989). These retrogradely labeled clustered neurons were distributed within the range from the bregma −12.72 to −12.00 mm, and no retrogradely labeled clustered neurons were found in the PHN rostral to the bregma −12.00 mm. Clustered PHN neurons labeled with Alexa Fluor 488 were not tomato-positive neurons, as observed in our previous study (Sugimura and Saito, 2021). The major neuron types in the PHN are glutamatergic, GABAergic, glycinergic, and cholinergic neurons (McCrea and Horn, 2006), and GABAergic and glycinergic neurons in the PHN do not directly project to the VC (Sugimura and Saito, 2021). Therefore, Alexa Fluor 488-labeled clustered PHN neurons that did not express tdTomato were determined to be glutamatergic. These results indicate that glutamatergic clustered preposito-lobule III–V neurons and preposito-crus I and II neurons are localized within the restricted ventral caudal region of the PHN.

Neurons in the medial vestibular nucleus, adjacent to the PHN, send mossy fiber projections to multiple cerebellar regions (Ando et al., 2020), suggesting that PHN neurons also send divergent projections to multiple cerebellar regions. To examine whether clustered preposito-lobule III–V and preposito-crus I and II neurons in the ventral caudal PHN project to both lobules III–V and crus I and II, we performed a dual-tracer injection experiment involving the injection of dextran-conjugated Alexa Fluor 488 into crus I and II in the cerebellar hemisphere and dextran-conjugated Alexa Fluor 594 into lobules III–V in the anterior vermis (*n* = 3; Fig. 1*h*). Figure 1*i* shows a ventral caudal PHN neuron that was double-labeled with Alexa Fluor 488 and Alexa Fluor 594 (arrow), indicating that this neuron projected to both crus I and II and lobules III–V. Figure 1*j* shows the proportions of double-labeled neurons among preposito-crus I and II neurons (10.2 ± 4.2%; *n* = 3 rats; total number of examined neurons, 39 ± 6) and preposito-lobule III–V neurons (7.3 ± 2.8%; *n* = 3 rats; total number of examined neurons, 56 ± 26) in the ventral caudal PHN. These results indicate that a small portion of preposito-crus I and II and preposito-lobule III–V neurons also project to lobules III–V and crus I and II, respectively.

### PHN neurons projecting to lobules III–V and crus I and II in the ventral caudal PHN did not project to the UN

We next assessed whether some preposito-lobule III–V or preposito-crus I and II neurons in the ventral caudal PHN project to the VC. We injected dextran-conjugated Alexa Fluor 488 into lobules III–V or crus I and II and dextran-conjugated Cascade Blue or Alexa Fluor 594 into the UN (Fig. 2*a*; *n* = 2; Fig. 2*d*; *n* = 2). As shown in Figure 1, Alexa Fluor 488-labeled clustered preposito-lobule III–V and preposito-crus I and II neurons were distributed between the bregma −12.72 and −12.00 mm, and no Alexa Fluor 488-labeled clustered neurons were observed in the PHN rostral to the bregma −12.00 mm (Fig. 2*b*,*e*). In contrast, Cascade Blue- or Alexa Fluor 594-labeled preposito-UN neurons were frequently observed in the PHN rostral to the bregma −12.00 mm but were rarely found in the PHN caudal to this level (Fig. 2*b*,*e*). We examined the Alexa Fluor 488-labeled clustered neurons (62 ± 17 neurons per rat; *n* = 4; from Fig. 2*a*,*d*) in the PHN between the bregma −12.72 and −12.00 mm and found that none of these neurons were double-labeled with Cascade Blue or Alexa Fluor 594 in addition to Alexa Fluor 488. These results indicate that preposito-lobule III–V and preposito-crus I and II neurons, which were localized in the ventral region of the caudal PHN, did not project to the UN.

### Electrophysiological properties of ventral caudal PHN neurons projecting to the anterior vermis and cerebellar hemisphere

We investigated the electrophysiological properties of PHN neurons in the ventral caudal PHN that project to the anterior vermis and the cerebellar hemisphere using whole-cell patch–clamp recordings. We recorded two types of neurons: (1) retrogradely labeled clustered neurons in the ventral caudal PHN after tracer injection into crus I and II and (2) unlabeled clustered neurons with large cell bodies in the ventral caudal PHN between the bregma −12.72 and −12.00 mm. The latter approach was based on our observation that neurons projecting to lobules III–V in the anterior vermis or crus I and II in the cerebellar hemisphere are consistently located in this region (Fig. 1*b*,*f*). This allowed us to target neurons for recording without retrograde labeling. The nucleus of the solitary tract (Fig. 1*b*,*f*) was used as a landmark to confirm that the brain slice location within the bregma was −12.72 to −12.00 mm.

**Figure 3.**
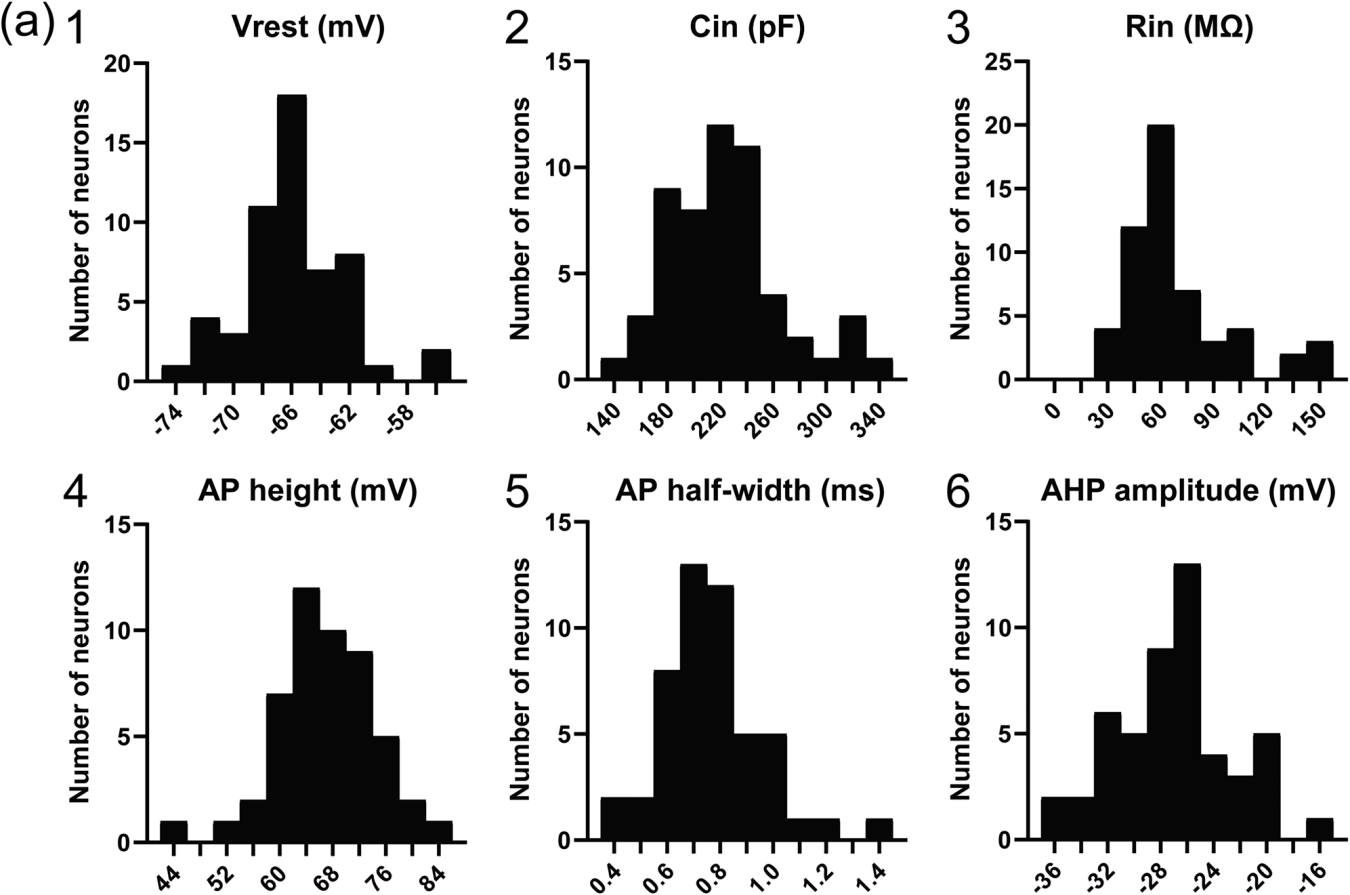
Histograms of the distributions of intrinsic electrophysiological properties of recorded neurons in the ventral caudal PHN projecting to the anterior vermis and cerebellar hemisphere. ***a*1–6**, Histograms showing the distributions of Vrest, Cin, Rin, AP height, AP half-width, and AHP amplitude of recorded neurons in the ventral caudal PHN, respectively: Vrest (−65.8 ± 3.6 mV; *N* = 55), Cin (224.5 ± 43.9 pF; *N* = 55), Rin (69.4 ± 30.7 MΩ; *N* = 55), AP height (67.1 ± 7.3 mV; *N* = 50), AP half-width (0.77 ± 0.19 ms; *N* = 50), and AHP amplitude (−27.0 ± 4.3 mV; *N* = 50). Vrest, resting membrane potential; Cin, input capacitance; Rin, input resistance; AP height, action potential height; AP half-width, action potential half-width; AHP amplitude, afterhyperpolarization amplitude.

**Figure 4.**
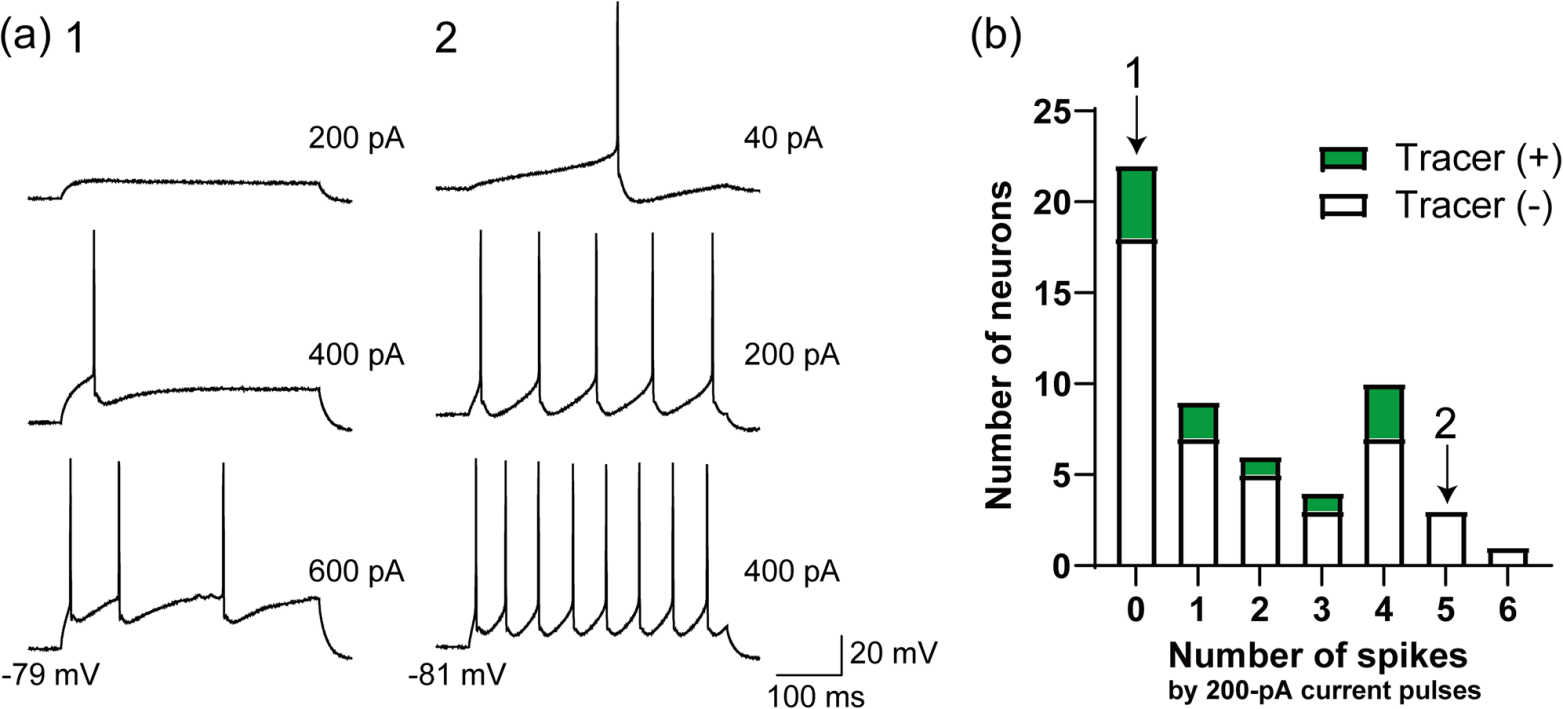
Low-frequency firing of most recorded neurons in response to depolarizing current pulses. ***a***, Representative firing traces of two neurons in the ventral caudal PHN in response to 400 ms depolarizing current pulses. Voltage traces are shown for three different current pulses. ***b***, A histogram showing the distribution of the number of spikes in neurons of the ventral caudal PHN in response to 200 pA current pulses. Data from tracer (+) and tracer (−) represent retrogradely labeled neurons in the ventral caudal PHN following tracer injection into crus I and II and neurons in the same region in the absence of tracer injection, respectively.

Figure 3 shows histograms of the distributions of the intrinsic electrophysiological properties of the recorded neurons. These histograms exhibit a unimodal distribution, suggesting that the recorded neurons form a population with similar intrinsic electrophysiological properties. Notably, the recorded PHN neurons did not fire spontaneously at their resting membrane potential. Our previous studies demonstrated that PHN neurons exhibit various firing patterns in response to depolarizing current pulses (Shino et al., 2008, 2011; Saito and Yanagawa, 2013, 2017; Zhang et al., 2014; Saito et al., 2015). Therefore, we next examined the firing patterns of the recorded neurons in the ventral caudal PHN. Figure 4*a* shows firing traces of two representative neurons, and Figure 4*b* presents a histogram of the spike count distribution for all recorded neurons in response to 200 pA current pulses. These results indicated that most of the neurons exhibited low-frequency firing despite sufficient membrane depolarization. This firing pattern is consistent with the LFR pattern previously characterized in our studies (Shino et al., 2008, 2011; Saito and Yanagawa, 2013, 2017; Zhang et al., 2014; Saito et al., 2015).

**Figure 5.**
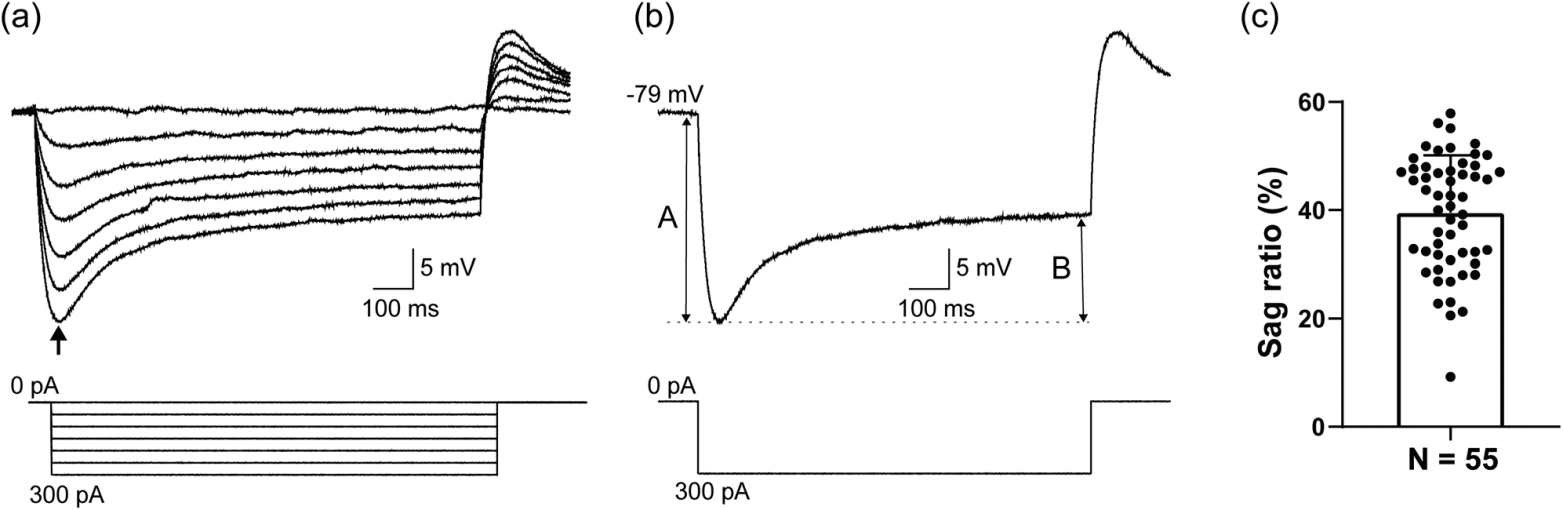
A prominent voltage sag in response to hyperpolarizing current pulses in recorded neurons. ***a***, Voltage responses of a neuron in the ventral region of the caudal PHN to hyperpolarizing current pulses (1 s duration, −50 pA steps). The arrow indicates the voltage sag, which is indicative of h-current activation. ***b***, Voltage response of the neuron in ***a*** to −300 pA current pulses. The ratio of B to A represents the sag ratio. ***c***, Sag ratio of neurons in the ventral region of the caudal PHN. Each plot shows data from individual neurons.

Furthermore, a subset of PHN neurons typically exhibits time-dependent inward rectification (voltage sag) caused by a hyperpolarization-activated cation current (Shino et al., 2008). Therefore, we investigated whether the recorded neurons in the ventral caudal PHN also exhibit this voltage sag. In response to hyperpolarizing current pulses, a representative neuron recorded in the ventral caudal PHN showed prominent voltage sag (Fig. 5*a*). Figure 5*c* shows a graph of the sag ratio for the recorded neurons. This analysis revealed that all the neurons examined exhibited voltage sag.

### Morphological characteristics of ventral caudal PHN neurons projecting to the anterior vermis and cerebellar hemisphere

**Figure 6.**
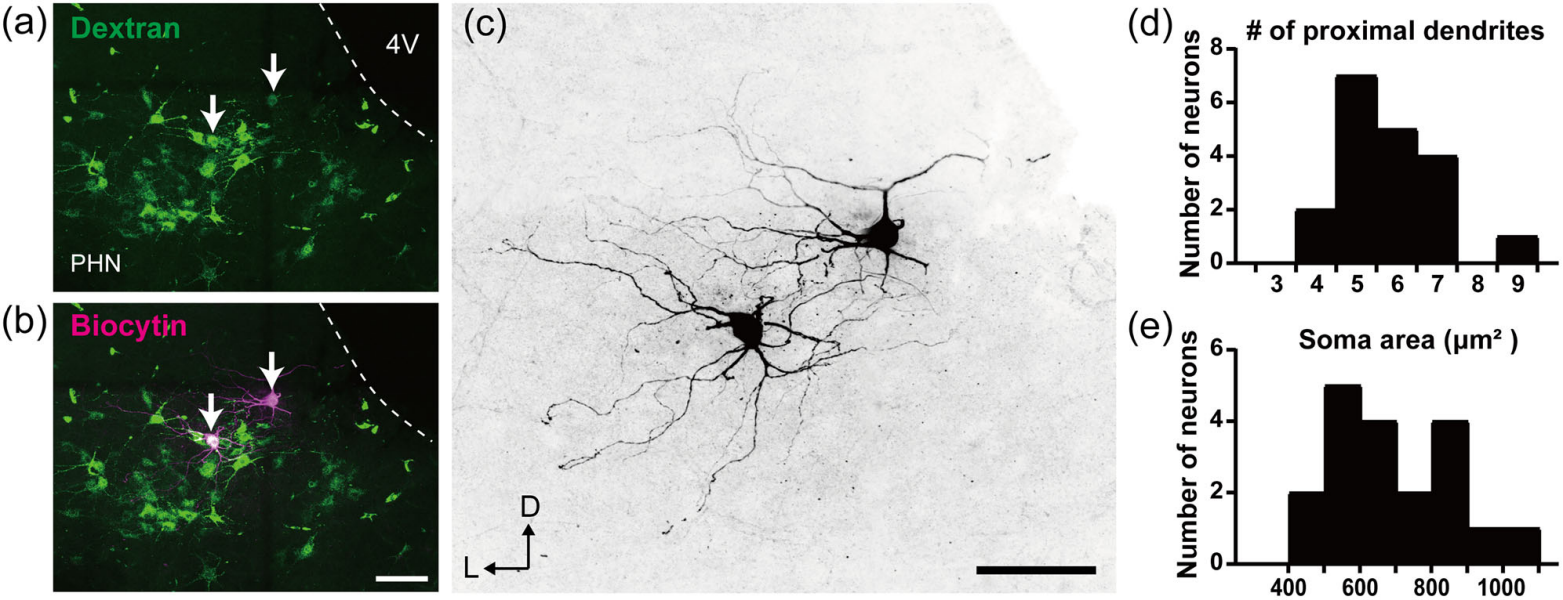
Morphological characteristics of biocytin-injected ventral caudal PHN neurons. ***a***, *Z*-stack confocal image of a frontal section containing the PHN, showing retrogradely labeled neurons following an injection of dextran-conjugated Alexa Fluor 488 into crus I and II. ***b***, Merged *Z*-stack confocal image of (***a***) PHN neurons stained with biocytin. The arrows indicate PHN neurons that were successfully injected with biocytin and visualized. ***c***, Enlarged image of the PHN neurons stained with biocytin in ***b***, corresponding to the neurons indicated by arrows. ***d***, ***e***, Histograms showing the distributions of the number of proximal dendrites, defined as dendrites extending directly from the soma (***d***) and the soma area of biocytin-injected ventral caudal PHN neurons (***e***). Scale bars, (***b***, ***c***) 100 μm.

We analyzed the morphological profiles of recorded neurons that were successfully injected with biocytin and whose somata and dendrites were clearly visualized (*n* = 19; 4 retrogradely labeled neurons following tracer injection and 15 unlabeled ventral caudal PHN neurons). The arrows in Figure 6,*a* and *b*, show retrogradely labeled neurons in the ventral caudal PHN following tracer injection into crus I and II. These neurons exhibited a multipolar shape characterized by dendrites extending in multiple directions (Fig. 6*c*). All the analyzed neurons displayed this multipolar morphology (Fig. 6*d*; *n* = 19), with an average number of proximal dendrites of 5.8 ± 1.2. Additionally, the soma area of the neurons showed a broad distribution, with a median area of 648.7 μm^2^ (Fig. 6*e*; *n* = 19).

## Discussion

The PHN exhibits major cytological features that are common across mammals, and distinct regional differences exist within it, with the caudal part being divided into a dorsolateral region (the parvocellular region of the PHN, PHs) and a ventromedial region (the magnocellular PHN, PHm) (McCrea and Horn, 2006). While the PHs contains small neurons, the PHm predominantly contains neurons with large cell bodies (Gaál et al., 2015) and multiple dendritic features, such as multiple, thick dendritic trees that radiate within the PHm (McCrea and Baker, 1985b; McCrea and Horn, 2006). In the macaque PHN, the PHm is no longer identifiable midway between the hypoglossal and abducens nuclei, which roughly corresponds to the midpoint along the rostrocaudal axis of the PHN, and it is absent rostral to this point (McCrea and Horn, 2006). Our study revealed that glutamatergic clustered neurons in the ventral region of the caudal PHN, which project to lobules III–V in the anterior vermis and crus I and II in the cerebellar hemisphere, exhibit a multipolar dendritic morphology and are located between the bregma −12.72 and −12.00 mm. These findings suggest that the clustered neurons examined in this study constitute a subset of the PHm cell group.

Previous studies on PHm–cerebellar projections have demonstrated that most PHm neurons, which are multidendritic cells in the ventral caudal PHN, project to the flocculus and nodulus (Brodal and Brodal, 1983; McCrea and Baker, 1985a; Røste, 1989; McCrea and Horn, 2006), whereas reports on their projections to cerebellar regions outside the VC are extremely limited, with the exception of the study of Røste (1989), who reported no projections to the cerebellar vermis and only sparse projections to crus I and II in cats. These findings suggest that PHm has limited interactions with cerebellar regions outside the VC. However, our study provides evidence that PHm neurons also project to cerebellar regions other than the VC, such as lobules III–V in the vermis and crus I and II in the cerebellar hemisphere. Additionally, we found that these PHm neuron groups do not project to the UN. Thus, in contrast to the previously recognized PHm neurons that project to the VC, we identified a specific subgroup of PHm neurons that project to the anterior vermis and the cerebellar hemispheres. These findings suggest that the PHm is not only involved in vestibulocerebellar functions but also may play a role in functional interactions with the cerebellar vermis and hemispheres.

Although we did not perform dual-tracer injections into the flocculus, vermis, or hemisphere in this study, previous anatomical data suggest that a substantial overlap between PHN neurons projecting to the flocculus and those in the ventral caudal PHN projecting to lobules III–V and crus I and II is unlikely. A previous study (Sugimura and Saito, 2021) reported that flocculus-projecting PHN neurons did not form a clustered population in the ventral caudal region, whereas the PHN neurons identified in the present study formed clearly clustered populations in this region. This anatomical distinction implies that the two groups are likely to represent distinct populations. Nevertheless, the possibility of partial overlap cannot be entirely excluded and should be examined in future studies.

The PHN and the VC are known to collaborate in controlling horizontal gaze, and PHN neurons projecting to the VC are thought to contribute to this function (Robinson, 1974; Zee et al., 1981; Kheradmand and Zee, 2011; Leigh and Zee, 2015). Furthermore, our recent study suggested that some PHN neurons serve as relay neurons in a neural pathway from the interstitial nucleus of Cajal (INC) to the VC and that these specific PHN neurons projecting to the VC may play a role in vertical gaze holding (Sugimura et al., 2024). In contrast to the functions of PHN neurons projecting to the VC, preposito-lobule III–V neurons and preposito-crus I and II neurons in the ventral caudal PHN have not been extensively studied, and their functions remain unknown. Multidendritic neurons within the PHm do not appear to send axons or collaterals to brainstem regions other than the inferior olive (McCrea and Horn, 2006). These findings suggest that the preposito-lobule III–V and preposito-crus I and II neurons in the PHm may function primarily as relay neurons that receive input from specific brain regions and transmit information to the cerebellar vermis and hemispheres. The cerebellar vermis and hemispheres are involved not only in motor coordination but also in emotion and cognitive functions (Bobée et al., 2000; Strick et al., 2009; Al-Afif et al., 2013; Wang et al., 2014; Sokolov et al., 2017; Schmahmann, 2019). Both cerebellar regions play distinct roles from those of the VC, as the VC primarily participates in ocular motor control and postural balance (Kheradmand and Zee, 2011; Leigh and Zee, 2015; Beh et al., 2017; Kandel et al., 2021). Therefore, preposito-lobule III–V and preposito-crus I and II neurons in the ventral caudal PHN may contribute to motor control beyond ocular motor function and postural balance, as well as nonmotor functions such as emotion and cognition.

Our electrophysiological study revealed that preposito-lobule III–V and preposito-crus I and II neurons exhibit high input capacitance, low input resistance, and an LFR pattern. In our previous study (Saito and Yanagawa, 2017), some neurons that exhibited an LFR pattern, along with the highest input capacitance and the lowest input resistance among all firing pattern types, were sampled from the PHN, excluding the extreme rostral and caudal ends. Although the firing patterns of preposito-lobule III–V and preposito-crus I and II neurons are similar to those of LFR neurons, preposito-lobule III–V and preposito-crus I and II neurons are glutamatergic, whereas most LFR neurons are cholinergic. This comparison was solely based on similarities in firing patterns and was not intended to suggest a match in neurotransmitter identity or projection targets. A previous study (Sugimura and Saito, 2021) reported that cholinergic neurons represent only a minor subset of PHN neurons that innervate the VC. In our study, we examined glutamatergic neurons projecting to lobules III–V and crus I and II (without the assumption that VC-projecting PHN neurons are predominantly cholinergic). Rather, the reference to LFR-type cells served to underscore the distinct electrophysiological features of the neurons that we identified, with the use of firing behavior as a point of contrast. Furthermore, the input capacitance (224.5 ± 43.9 pF; *N* = 55) and the input resistance (69.4 ± 30.7 MΩ; *N* = 55) of the preposito-lobule III–V and preposito-crus I and II neurons were greater than twice the capacitance (95.9 ± 37.8 pF; *N* = 22; *p* < 0.0001; unpaired *t* test) and approximately one-fifth the resistance (372.8 ± 198.2 MΩ; *N* = 22; *p* < 0.0001; Welch's *t* test) of LFR neurons. Therefore, preposito-lobule III–V and preposito-crus I and II neurons are electrophysiologically distinct not only from LFR neurons but also from all other PHN neuron types reported in that study. The extremely low values of input resistance of preposito-lobule III–V and preposito-crus I and II neurons imply that the membrane potential of these neurons is less susceptible to fluctuations in response to input currents. In addition, none of the recorded PHN neurons in this study exhibited spontaneous firing at their resting membrane potential, although other PHN neurons typically exhibit spontaneous firing (Shino et al., 2011; Saito and Yanagawa, 2013). These findings suggest that preposito-lobule III–V and preposito-crus I and II neurons, in contrast to other PHN neurons, require stronger synaptic input to generate action potentials and therefore cannot transmit signals to the cerebellum unless they receive strong, synchronized synaptic input. In contrast to these firing characteristics, PHN neurons projecting to abducens motoneurons and interneurons exhibit slow adaptation and the capacity to sustain relatively high-frequency firing (Navarro-López et al., 2004), which is crucial for encoding horizontal eye position. In the present study, ventral caudal PHN neurons projecting to the anterior vermis and cerebellar hemisphere consistently exhibited low-frequency firing, even under sufficient depolarization. This marked difference in firing behavior suggests distinct input–output relationships and functional roles between these two PHN neuron populations. The inability to sustain high-frequency discharge, which is a key feature required for eye position encoding, further supports the notion that ventral caudal PHN neurons projecting to the cerebellum constitute a functionally distinct subpopulation from those involved in oculomotor control via abducens projections. The lower input resistance may be partially explained not only by the large size of the cells but also by the involvement of hyperpolarized-activated cyclic nucleotide-gated (HCN) channels. In a previous study, we reported that Hcn1 knock-out rats exhibit increased input resistance in cortical and CA1 pyramidal neurons compared with wild-type rats (Nishitani et al., 2019), indicating that neurons expressing HCN channels have reduced input resistance. In this study, all preposito-lobule III–V and preposito-crus I and II neurons exhibited prominent voltage sag, suggesting HCN channel involvement in their electrical behavior. Therefore, the low input resistance observed in these neurons is at least partially attributable to the presence of HCN channels.

While PHN neurons are known to constitute a heterogeneous population with diverse electrophysiological, anatomical, and morphological properties (McCrea and Baker, 1985b; Idoux et al., 2006 ; McCrea and Horn, 2006; Shino et al., 2008; Kolkman et al., 2011; Zhang et al., 2014; Gaál et al., 2015; Saito et al., 2015; Saito and Yanagawa, 2017), our study demonstrated that the clustered preposito-lobule III–V and preposito-crus I and II neurons in the ventral region of the caudal PHN form a homogeneous neuronal population in terms of each of these properties. Electrophysiologically, these neurons exhibited similar firing patterns, intrinsic properties, and HCN channel expression. Anatomically and morphologically, they showed similar localization in the PHN and had a multipolar morphology. In contrast, their features were distinct from those of other PHN neurons, with unique cerebellar projection sites and specific electrophysiological properties. These findings suggest that the clustered preposito-lobule III–V and preposito-crus I and II neurons in the ventral caudal PHN identified in this study likely represent a functionally distinct subpopulation within the PHN. While previous research has emphasized PHN projections to the VC and their role in ocular motor control, our study presents new evidence suggesting that a distinct PHN subpopulation contributes to broader motor control functions through interactions with the cerebellar vermis and hemispheres.

## Synthesis

Reviewing Editor: David Schoppik, New York University Grossman School of Medicine Decisions are customarily a result of the Reviewing Editor and the peer reviewers coming together and discussing their recommendations until a consensus is reached. When revisions are invited, a fact-based synthesis statement explaining their decision and outlining what is needed to prepare a revision will be listed below. The following reviewer(s) agreed to reveal their identity: José María Delgado-García.

Major

1. It's already known that the PHN projects to several regions of the cerebellum outside the VC and is expected to function in broader motor control. We found the current Abstract and Significant Statement are a bit misleading as these statements are not originated from the current work. Is the implication that the cholinergic neurons are mostly dedicated to eye position but the glutamatergic neurons appear to be separated from those neurons and responsible for other functional domains? Could any implications be drawn from the intrinsic properties in those broader functions? If so, please rephrase Abstract and Significant Statement to highlight these points.

2. We found the interpretation of pre-VC neurons as cholinergic neurons in the Discussion to be confusing. This appears contradictory to Sugimura and Saito (2021, J Comp Neurol), which demonstrates that cholinergic neurons constitute only a minor subset of pre-VC neurons. The authors compared the intrinsic properties between the pre-other neurons obtained in this study and the cholinergic neurons obtained previously. Consequently, the electrophysiological cell type distinctions identified by the authors likely represent differences between cholinergic vs glutamatergic precerebellar neurons, rather than between pre-VC vs pre-other cerebellar neurons. A critical experiment comparing pre-VC glutamatergic neurons is missing from this study, which limits the validity of their conclusions about pre-VC vs pre-other. The comparison is further complicated by the demonstration that cholinergic neurons could project to the regions other than pre-VC region (Sugimura and Saito (2021, J Comp Neurol)) -- in this case, recording from pre-other cholinergic neurons would be required. Please clarify. This confusion also impacts the clarity of the Abstract.

3. The authors used dual injections involving UN to argue that pre-other PHN neurons are distinct from pre-VC neurons. However, among the pre-VC neurons (pre-uvula/nodulus and pre-flocculus), it's known that the uvula/nodulus projecting PHN neurons are largely separated from the flocculus projecting PHN neurons (Tan and Gerrits, 1992, Neuroscience). It is still possible that pre-other neurons are the same as pre-flocculus neurons. Therefore, authors' conclusion could not be supported without performing dual injections to flocculus and vermis, and to flocculus and hemisphere.

4. Introduction is missing introductions to help understand the electrophysiological experiments. For example, what are the physiological classification of PHN neurons? What are the LFR neurons?

5. The paper deals with an interesting population of neurons not reported yet, but fails to explain its physiological function. Specifically, the authors did not address the study of their physiological functions (neck or tongue movements) by recording their firing properties in alert behaving rats and their proper identification by their antidromic activation from their projection sites.

6. The manuscript would benefit from a mention in the Introduction of the important population of PH neurons projecting to the abducens nucleus carrying eye velocity and eye position signals (see, for example, Delgado-Garcia et al., Neuroscience, 1989; Escudero et al., Journal of Physiology, London, 1992).

7. It could be also interesting the comparison of the firing properties of the population of cells described in the present study with those presented by PH neurons projecting to abducens motoneurons and interneurons (see Navarro-Lopez et al., Journal of Neuroscience, 2004). These different firing properties could be commented in the Discussion section.

Minor

1. Please indicate in the Abstract that this study used rats, if the authors wish to include Bregma coordinates in the Abstract.

2. Photos of the injections to Crus are too small to identify whether the injections are localized to the target. It appears they spread to other regions-- in this case, crus-projecting neurons as appear in Abstract would be an overstatement.

## Author Response

Synthesis of Reviews:

Computational Neuroscience Model Code Accessibility Comments for Author (Required):

N/A Synthesis Statement for Author (Required):

Major 1. It's already known that the PHN projects to several regions of the cerebellum outside the VC and is expected to function in broader motor control. We found the current Abstract and Significant Statement are a bit misleading as these statements are not originated from the current work. Is the implication that the cholinergic neurons are mostly dedicated to eye position but the glutamatergic neurons appear to be separated from those neurons and responsible for other functional domains? Could any implications be drawn from the intrinsic properties in those broader functions? If so, please rephrase Abstract and Significant Statement to highlight these points.

We appreciate the reviewer's comment and acknowledge that projections from the PHN to cerebellar regions located outside of the vestibulocerebellum (VC), as well as their potential roles in broader motor control, have been previously reported. Previous studies have demonstrated that most PHN neurons in the ventral caudal PHN (the region where we have previously reported the existence of a unique neuronal population, which is the subject of this study) project to the flocculus and nodulus (Brodal and Brodal, 1983; R. A. McCrea and Baker, 1985; Røste, 1989; McCrea and Horn, 2006), whereas another study (Røste, 1989) reported that neurons in the ventral caudal PHN sparsely project to crus I-II in cats and do not project to the cerebellar anterior vermis. These findings suggested that the ventral caudal PHN did not significantly interact with the cerebellar anterior vermis or hemispheres. To our knowledge, this is the first study to thoroughly characterize the localization, electrophysiological, and morphological properties of a subpopulation of neurons in the ventral caudal PHN that project to the cerebellar anterior vermis and hemisphere. To avoid any misunderstanding that PHN projections to non-VC cerebellar regions or their involvement in broader motor control represent novel findings of this study, we have revised the Significance Statement and part of the Abstract accordingly.

The Significance Statement (page 2, lines 7-14) now reads: "This study examined a subpopulation of neurons in the ventral caudal PHN that project to the cerebellar anterior vermis and hemisphere, with the properties of these neurons having not been previously characterized. We demonstrated that these neurons exhibit distinct localization, electrophysiological, and morphological properties, thus suggesting that they are different from previously characterized PHN neurons. Our findings suggest that this PHN subpopulation (via its interactions with the cerebellar vermis and hemispheres) may contribute to broader motor control functions beyond oculomotor control and highlight the functional heterogeneity within the PHN." In the Abstract, we changed "The prepositus hypoglossi nucleus (PHN) is involved in horizontal gaze control. Although the PHN functions by cooperating with the vestibulocerebellum (VC), there are few reports on PHN projections to cerebellar regions outside the VC." to "The prepositus hypoglossi nucleus (PHN), involved in horizontal gaze control, contributes to this function via cooperation with the vestibulocerebellum (VC). Furthermore, some PHN neurons have also been observed to project to cerebellar regions outside the VC." (page 1, lines 2-5) to better reflect previous findings.

We did not intend to imply the existence of such a functional segregation based on neurotransmitter type. The mention of cholinergic PHN neurons was solely included within the context of providing an electrophysiological comparison to better understand the intrinsic properties of the glutamatergic neurons that we recorded. We compared our identified neurons with the low firing rate pattern (LFR) subtype that was classified in a previous study (Saito and Yanagawa, 2017). Due to the fact that both types of neurons exhibited similarities in their basic firing patterns, we considered it appropriate to use LFR neurons as a reference for highlighting electrophysiological distinctions (rather than other PHN neuron subtypes with different firing patterns). Our statement that LFR neurons are primarily cholinergic was included simply to provide additional known characteristics of this subtype and was not intended to imply a classification or functional segregation based on neurotransmitter type. Our comparison was simply focused on intrinsic electrophysiological properties. To avoid redundancy, we have provided a more detailed explanation of this point in our response to Comment 2 and have also clarified this issue in the revised Discussion section (from page 24, lines 9-16).

Although directly inferring specific functions solely from intrinsic electrophysiological properties is beyond the scope of the present study (which focused on characterization analysis rather than functional analysis), these properties may still provide valuable clues. As similarly discussed in our response to Comment 7, the neurons that we recorded exhibited consistent low-frequency firing even under sufficient depolarization. This is in contrast to the high-frequency firing capability that is crucial for encoding horizontal eye position, as demonstrated by PHN neurons projecting to the abducens nucleus (Navarro-Lopez et al., 2004). This notable difference in intrinsic excitability and firing behavior suggests that the PHN subpopulation projecting to non-VC regions represents a functionally distinct group that is likely involved in additional motor control other than eye position encoding, thereby supporting their potential role in broader motor functions. This point has been added to the revised Discussion section (from page 25, line 5 from the bottom to page 26, line 7).

We have also added the corresponding references (Navarro-López et al., 2004) to the reference list.

2. We found the interpretation of pre-VC neurons as cholinergic neurons in the Discussion to be confusing. This appears contradictory to Sugimura and Saito (2021, J Comp Neurol), which demonstrates that cholinergic neurons constitute only a minor subset of pre-VC neurons. The authors compared the intrinsic properties between the pre-other neurons obtained in this study and the cholinergic neurons obtained previously. Consequently, the electrophysiological cell type distinctions identified by the authors likely represent differences between cholinergic vs glutamatergic precerebellar neurons, rather than between pre-VC vs pre-other cerebellar neurons. A critical experiment comparing pre-VC glutamatergic neurons is missing from this study, which limits the validity of their conclusions about pre-VC vs pre-other. The comparison is further complicated by the demonstration that cholinergic neurons could project to the regions other than pre-VC region (Sugimura and Saito (2021, J Comp Neurol)) -- in this case, recording from pre-other cholinergic neurons would be required. Please clarify. This confusion also impacts the clarity of the Abstract.

First, we would like to clarify that our study neither interprets nor assumes that pre-VC neurons are predominantly cholinergic. As correctly noted by the reviewer and consistent with the results of Sugimura and Saito (2021, J Comp Neurol), cholinergic neurons constitute only a minor subset of the pre-VC neuron population. The primary aim of our study was not to compare cholinergic and glutamatergic neurons; rather, we aimed to characterize the electrophysiological and morphological properties of glutamatergic clustered preposito-lobule III-V and preposito-crus I-II neurons in the ventral caudal PHN, as identified in this study.

In the Discussion, we referred to the study by Saito and Yanagawa (2017, Neurosci Res) to compare the electrophysiological properties of our recorded neurons. We compared them with the low firing rate pattern (LFR) subtype of PHN neurons, which are primarily cholinergic and exhibit a similar firing pattern among the six firing types that were classified in that study. We selected this comparison because the comparison of neurons with similar firing patterns is a reasonable approach for highlighting electrophysiological differences; moreover, differences in firing patterns are typically accompanied by differences in intrinsic electrophysiological properties.

According to Saito and Yanagawa (2017, Neurosci Res), LFR neurons exhibit the lowest input resistance (Ri) and the highest input capacitance (Cin) among the six classified firing types. In contrast, our recorded neurons exhibited an even lower Ri and higher Cin compared with LFR neurons, thus indicating that they are electrophysiologically distinct not only from LFR neurons but also from all of the other PHN neuron types that were reported in that study.

Furthermore, Zhang et al. (2014, Eur. J. Neurosci.) demonstrated that even cholinergic PHN neurons represent a heterogeneous population, exhibiting diverse firing patterns and electrophysiological properties. These findings support our rationale for focusing on firing behavior (rather than neurotransmitter types or projection targets) in our comparisons. Thus, our reference to cholinergic LFR neurons was solely intended to highlight the distinct electrophysiological characteristics of the glutamatergic neurons that we identified by contrasting them with a previously characterized subtype.

However, the primary objective of this study was to characterize a previously unrecognized population of clustered preposito-lobule III-V and preposito-crus I-II neurons in the ventral caudal PHN. Therefore, direct comparisons with either pre-VC glutamatergic neurons or cholinergic neurons projecting to non-VC regions were beyond the scope of the present research. Nonetheless, such comparisons would be valuable for clarifying electrophysiological distinctions among PHN subtypes, and future studies addressing these questions will be important for refining their classification and functional interpretation.

In response to the reviewer's insightful comment, we have incorporated the abovementioned discussion points into three specific sections of the Discussion section (page 24, lines 4-5; page 24, lines 9-16; and page 25, lines 3-5).

3. The authors used dual injections involving UN to argue that pre-other PHN neurons are distinct from pre-VC neurons. However, among the pre-VC neurons (pre-uvula/nodulus and pre-flocculus), it's known that the uvula/nodulus projecting PHN neurons are largely separated from the flocculus projecting PHN neurons (Tan and Gerrits, 1992, Neuroscience). It is still possible that pre-other neurons are the same as pre-flocculus neurons. Therefore, authors' conclusion could not be supported without performing dual injections to flocculus and vermis, and to flocculus and hemisphere.

We agree that previous findings (Tan and Gerrits, 1992) indicate a clear separation between uvula/nodulus-projecting and flocculus-projecting PHN neurons. As the reviewer suggested, this raises the possibility that some of the pre-other neurons observed in our study may actually correspond to pre-flocculus neurons. We acknowledge that our study did not include dual-tracer injections into the flocculus, vermis or hemisphere. Therefore, we cannot fully exclude the possibility of overlap between these neuronal populations, which represents a limitation of our current approach.

However, based on the anatomical data from Sugimura and Saito (2021, J Comp Neurol), there is supportive evidence for distinguishing these populations. These researchers reported that flocculus-projecting PHN neurons do not form clustered populations in the ventral caudal PHN, whereas preposito-lobule III-V and preposito-crus I-II neurons are densely clustered in this region. This anatomical difference suggests that these two groups are largely distinct.

To address this concern regarding the potential overlap with flocculus-projecting PHN neurons, we have included the following paragraph in the revised Discussion (page 22, lines 1-10): "Although we did not perform dual-tracer injections into the flocculus, vermis or hemisphere in this study, previous anatomical data suggest that a substantial overlap between PHN neurons projecting to the flocculus and those in the ventral caudal PHN projecting to lobules III-V and crus I-II is unlikely. A previous study (Sugimura and Saito, 2021) reported that flocculus-projecting PHN neurons did not form a clustered population in the ventral caudal region, whereas the PHN neurons identified in the present study formed clearly clustered populations in this region. This anatomical distinction implies that the two groups are likely to represent distinct populations. Nevertheless, the possibility of partial overlap cannot be entirely excluded and should be examined in future studies." Furthermore, to more clearly reflect the distinction between the clustered ventral caudal PHN neurons that were identified in our study and those projecting to the flocculus, as well as to minimize potential misunderstandings regarding the target region, we have revised the manuscript as follows to improve clarity and accuracy:

In the Abstract (page 1, line 1 from the bottom), we now explicitly state that these PHN neurons "are likely to" constitute a distinct subpopulation from those projecting to the vestibulocerebellum (VC), in order to more clearly reflect the uncertainty regarding potential overlap with flocculus-projecting neurons.

In the Introduction (page 5, lines 5-6), we have clarified that the investigated projection site was specifically referred to as the uvula/nodulus (UN), rather than the use of the broader term "VC", in order to avoid potential misunderstandings regarding the target region.

4. Introduction is missing introductions to help understand the electrophysiological experiments. For example, what are the physiological classification of PHN neurons? What are the LFR neurons? In response, we have revised the Introduction accordingly. In the third paragraph (from page 5, lines 9-17), we now include a description of the electrophysiological classification of PHN neurons based on their characteristic firing patterns in response to depolarizing current injections. These firing patterns are classified into six distinct types, and we briefly mention the low firing rate (LFR) pattern as an example, which is characterized by a low number of spikes despite sufficient membrane depolarization. Furthermore, we explain the significance of this classification, noting that these firing patterns reflect unique intrinsic electrophysiological properties and that these patterns are associated with distinct input-output relationships. This information is essential for understanding the diversity of neuronal populations within the PHN.

In addition, due to the fact that the insertion of this lengthy explanation made the meaning of the following sentence less clear ("the PHN neurons that constitute the population"), we revised this sentence to "the preposito-lobule III-V and preposito-crus I-II neurons in the ventral caudal PHN" (page 5, lines 1-2 from the bottom) to improve clarity. Furthermore, to streamline the description, we revised "we investigated the electrophysiological and morphological properties of preposito-lobule III-V neurons and preposito-crus I-II neurons in the ventral caudal PHN" to "we investigated their electrophysiological and morphological properties" (page 6, line 1).

We have also added the corresponding references (Saito et al., 2012; Saito et al., 2017) to the reference list.

5. The paper deals with an interesting population of neurons not reported yet, but fails to explain its physiological function. Specifically, the authors did not address the study of their physiological functions (neck or tongue movements) by recording their firing properties in alert behaving rats and their proper identification by their antidromic activation from their projection sites.

We appreciate the reviewer's insightful comment. The present study did not aim to investigate the functional roles of these neurons in behaving animals, nor did it include antidromic identification from their projection sites. Our primary goal was to characterize the anatomical location, projection targets, and intrinsic electrophysiological and morphological properties of a specific population of PHN neurons projecting to cerebellar regions located outside of the vestibulocerebellum. These fundamental properties have not been previously described and are essential for understanding the potential diversity of PHN-cerebellar pathways.

Although the physiological functions of these neurons (including involvements in neck or tongue movements) remain to be determined, our findings provide a necessary foundation for future in vivo functional studies. We fully agree that such studies (particularly those involving neuronal recordings during specific behaviors and functional connectivity analyses via retrograde identification or stimulation) will be crucial for elucidating how this PHN subpopulation contributes to motor control. We intend to pursue these research directions in subsequent studies.

6. The manuscript would benefit from a mention in the Introduction of the important population of PH neurons projecting to the abducens nucleus carrying eye velocity and eye position signals (see, for example, Delgado-Garcia et al., Neuroscience, 1989; Escudero et al., Journal of Physiology, London, 1992).

Based on this comment, we have added a sentence to the Introduction to highlight the population of PHN neurons that project to the abducens nucleus and that are involved in conveying eye velocity and eye position signals. The revised sentence reads as follows (page 3, lines 3-6): "PHN neurons encode eye velocity and eye position signals (Delgado-Garcia et al., 1989; Escudero et al., 1992), and those neurons projecting to the abducens nucleus have been reported to primarily convey eye position signals (Escudero et al., 1992)." This addition helps to provide a more comprehensive background on the known functional projections of PHN neurons and their relevance to oculomotor control.

We have also added the corresponding references (Delgado-Garcia et al., 1989; Escudero et al., 1992) to the reference list.

7. It could be also interesting the comparison of the firing properties of the population of cells described in the present study with those presented by PH neurons projecting to abducens motoneurons and interneurons (see Navarro-Lopez et al., Journal of Neuroscience, 2004). These different firing properties could be commented in the Discussion section.

As the reviewer noted, Navarro-Lopez et al. (2004) reported that PH neurons projecting to the abducens nucleus exhibit slow adaptation and the capacity to sustain relatively high-frequency firing. This sustained high-frequency activity is essential for encoding horizontal eye position. In contrast, as investigated in the current study, the ventral caudal PHN neurons projecting to the anterior vermis and cerebellar hemisphere consistently exhibited low-frequency firing, even under sufficient depolarization. This marked difference in firing properties suggests the existence of distinct input-output relationships between these two PHN neuron populations. The inability to sustain high-frequency discharge, which is a key feature of eye position encoding, further supports the idea that ventral caudal PHN neurons projecting to the cerebellum represent a functionally distinct subpopulation compared with PHN neurons involved in oculomotor control via abducens projections. This comparison and discussion have been added to the revised manuscript in the Discussion section (page 25, line 5 from the bottom to page 26, line 7).

Minor 1. Please indicate in the Abstract that this study used rats, if the authors wish to include Bregma coordinates in the Abstract.

We have added "in rats" to the Abstract to clarify the species that was utilized in the current study (page 1, line 9), as follows: "Because the properties of these neurons have not been clarified, this study aimed to determine their localization, projections, and electrophysiological and morphological characteristics in rats." 2. Photos of the injections to Crus are too small to identify whether the injections are localized to the target. It appears they spread to other regions-- in this case, crus-projecting neurons as appear in Abstract would be an overstatement.

To clarify the location of the injection site, we have revised the schematic drawings to better match the rostrocaudal level of the injection site photomicrographs, as the previous schematic was slightly caudal. In the revised schematic, we have included the posterior superior fissure (PSF), which is an anatomical landmark that delineates the boundary between crus I-II and the adjacent simple lobule. The PSF is also marked on the fluorescence photomicrographs with dashed lines and labeled with arrows to aid in anatomical identification.

These revisions show that the vast majority of the fluorescence signal is located lateral to a vertical line drawn from the deepest point of the PSF, thereby indicating that the injection was well confined to crus I-II, with minimal (if any) spread to the surrounding areas. Although a small amount of fluorescence was observed medial to the PSF, this was most likely due to anterograde and/or retrograde transport of the tracer that was taken up at the injection site along white matter tracts associated with crus I-II (rather than indicating that the tracer had spread into neighboring cortical areas). Furthermore, no strong fluorescence was observed in other cerebellar lobules. Based on these observations, we have determined that the term "crus-projecting neurons" remains appropriate for inclusion in the Abstract, as the injection site was localized within crus I-II.

We chose not to enlarge the photomicrographs to preserve the visibility of the surrounding cerebellar regions and to demonstrate that the tracer did not spread to adjacent lobules, including both the vermal and hemispheric regions. To facilitate visual assessment despite the original image size being maintained, we have added dashed lines indicating the PSF and have clarified the rostrocaudal level in the revised schematic, which together allow readers to verify that the tracer was confined to crus I-II.

In addition, we have revised the figure legends to clarify the anatomical location of the injection site. On page 36 (lines 2-4 from the bottom), we added the following sentence: "The PSF is shown as an anatomical landmark in photomicrographs to indicate that the injection site was within crus I-II. In photos (e) and (h), the PSF is indicated by dashed lines." Additionally, on page 37 (lines 5-7 from the bottom), we included a similar sentence: "The PSF is shown as an anatomical landmark in the photomicrograph to indicate that the injection site was within crus I-II." The abbreviation "PSF, posterior superior fissure" was also defined on page 36 (lines 5-6 from the bottom) and on page 37 (lines 2-3 from the bottom).
